# Hydrological Response to Climate Change for Gilgel Abay River, in the Lake Tana Basin - Upper Blue Nile Basin of Ethiopia

**DOI:** 10.1371/journal.pone.0079296

**Published:** 2013-10-24

**Authors:** Yihun Taddele Dile, Ronny Berndtsson, Shimelis G. Setegn

**Affiliations:** 1 Stockholm Environment Institute, Stockholm, Sweden; 2 Stockholm Resilience Center, Stockholm University, Stockholm, Sweden; 3 Center for Middle Eastern Studies & Department of Water Resources Engineering, Lund University, Lund, Sweden; 4 Department of Earth and Environment, Florida International University, Miami, Florida, United States of America; University of Oxford, United Kingdom

## Abstract

Climate change is likely to have severe effects on water availability in Ethiopia. The aim of the present study was to assess the impact of climate change on the Gilgel Abay River, Upper Blue Nile Basin. The Statistical Downscaling Tool (SDSM) was used to downscale the HadCM3 (Hadley centre Climate Model 3) Global Circulation Model (GCM) scenario data into finer scale resolution. The Soil and Water Assessment Tool (SWAT) was set up, calibrated, and validated. SDSM downscaled climate outputs were used as an input to the SWAT model. The climate projection analysis was done by dividing the period 2010-2100 into three time windows with each 30 years of data. The period 1990-2001 was taken as the baseline period against which comparison was made. Results showed that annual mean precipitation may decrease in the first 30-year period but increase in the following two 30-year periods. The decrease in mean monthly precipitation may be as much as about -30% during 2010-2040 but the increase may be more than +30% in 2070-2100. The impact of climate change may cause a decrease in mean monthly flow volume between -40% to -50% during 2010-2040 but may increase by more than the double during 2070-2100. Climate change appears to have negligible effect on low flow conditions of the river. Seasonal mean flow volume, however, may increase by more than the double and +30% to +40% for the Belg (small rainy season) and Kiremit (main rainy season) periods, respectively. Overall, it appears that climate change will result in an annual increase in flow volume for the Gilgel Abay River. The increase in flow is likely to have considerable importance for local small scale irrigation activities. Moreover, it will help harnessing a significant amount of water for ongoing dam projects in the Gilgel Abay River Basin.

## Introduction

Climate change will increase the number of people living in water stressed regions globally [1,2]. The impact will be worse for the contemporary African population where about 25% already experience water stress [3]. Considering population increase and water use it has been estimated that the portion of the African population at risk for water stress and scarcity will increase to 65% in 2025 [4]. Climate change is, however, expected to exacerbate the current stress on water resources availability in Africa [1]. 

In Ethiopia 84% of the population base their daily living from agricultural production [5]. Agriculture in Ethiopia accounts for 47% of the GDP, 90% of exports, and 85% of employment [6]. The country’s economic policy is agriculturally based industrialization to reduce poverty and generate economic development. The heavy reliance of the Ethiopian economy on rainfed subsistence agriculture makes it particularly vulnerable to hydrological variability [7]. Moreover climate change may further reinforce the vulnerability of agriculture by increasing rainfall variability and evapotranspiration losses [8–11].

Previous research has shown that the water resources of Ethiopia are highly sensitive to climate change and variability [7,12–17]. Appropriate adaptation strategies are important policy options to limit the unprecedented impact of climate change for the livelihoods of the rural Ethiopian poor [8,17]. While the focus on considering global impact of climate change is primarily on societal responses to the local and regional consequences of large-scale changes [18], most climate change studies in Ethiopia have been done either at country or river basin scale. Therefore, results from these studies (e.g.[12–15,19]) are highly aggregated and have little importance in informing the impact of climate change at smaller scale (*cf.* [20,21]). The present research assesses the impact of climate change for the Gilgel Abay River, one of the major tributaries of the Lake Tana – the source of the Upper Blue Nile River. The Gilgel Abay River and the Lake Tana are important for various socio-economic purposes. However, due to climate change and variability, the water level in the lake fluctuates. Different studies (e.g., [22–24]) have quantified the impact of climate change on the Gilgel Abay and the Lake Tana Basin. Our study extends the understanding of the implications of climate change at the Gilgel Abay River by applying a process-based hydrological model with finer temporal and spatial resolution. Process-based hydrological models have a strong physical foundation for quantifying climate change impact [25]. Therefore, this study can potentially provide valuable insight to decision makers on the local vulnerability of the Gilgel Abay River and the Lake Tana regarding future change in rainfall and temperature because of climate change. Moreover, the methodology presented in this paper can be a useful approach to study the impact of climate change on the water resources of other basins.

## Materials and Methods

### Study area

**Figure 1:**
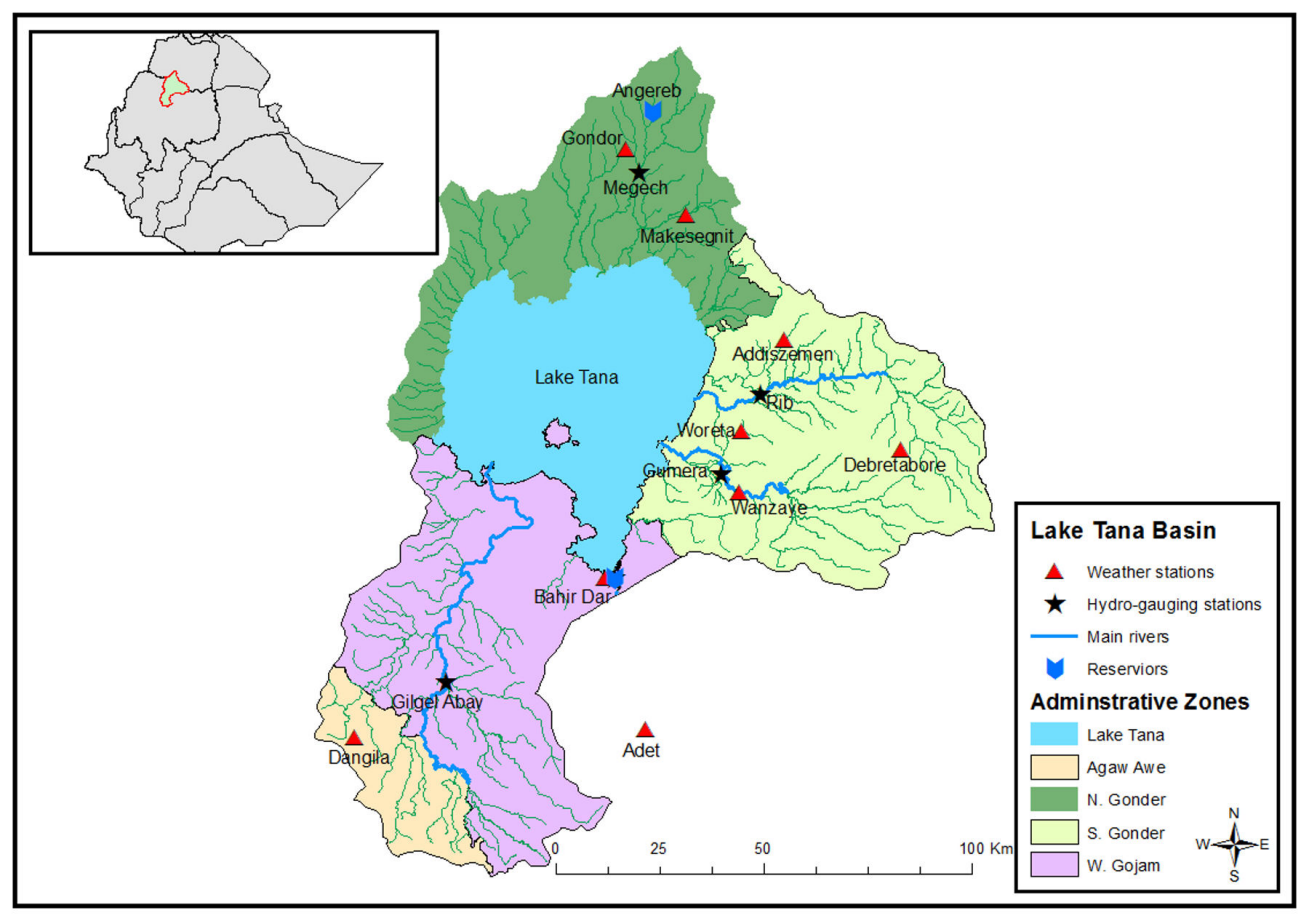
The location of the Lake Tana basin in the Ethiopian and the Upper Blue Nile Basin system with meteorological and river gauging station locations.

The Lake Tana Basin is located in northwestern Ethiopia (latitude 10.95° and 12.78°N, and longitude 36.89° and 38.25°E) with a drainage area of about 15,000 km^2^ [26] (Figure 1). It is shared by four administrative zones called Agew Awi, North Gondor, South Gondor, and West Gojjam. The Lake Tana, the largest lake in Ethiopia and the third largest in the Nile Basin, is located in this basin. The climate of the Lake Tana sub-basin is dominated by tropical highland monsoon with most of its rainfall (70-90% of total rainfall) occurring between June and September [10,27]. The major rivers feeding the Lake Tana are Gilgel Abay, Gumara, Ribb, and Megech. These rivers contribute more than 93% of the flow [28]. The Gilgel Abay River with a catchment area of 5,004 km^2^ is the largest river discharging into the Lake Tana. 

### Modeling approach

**Figure 2:**
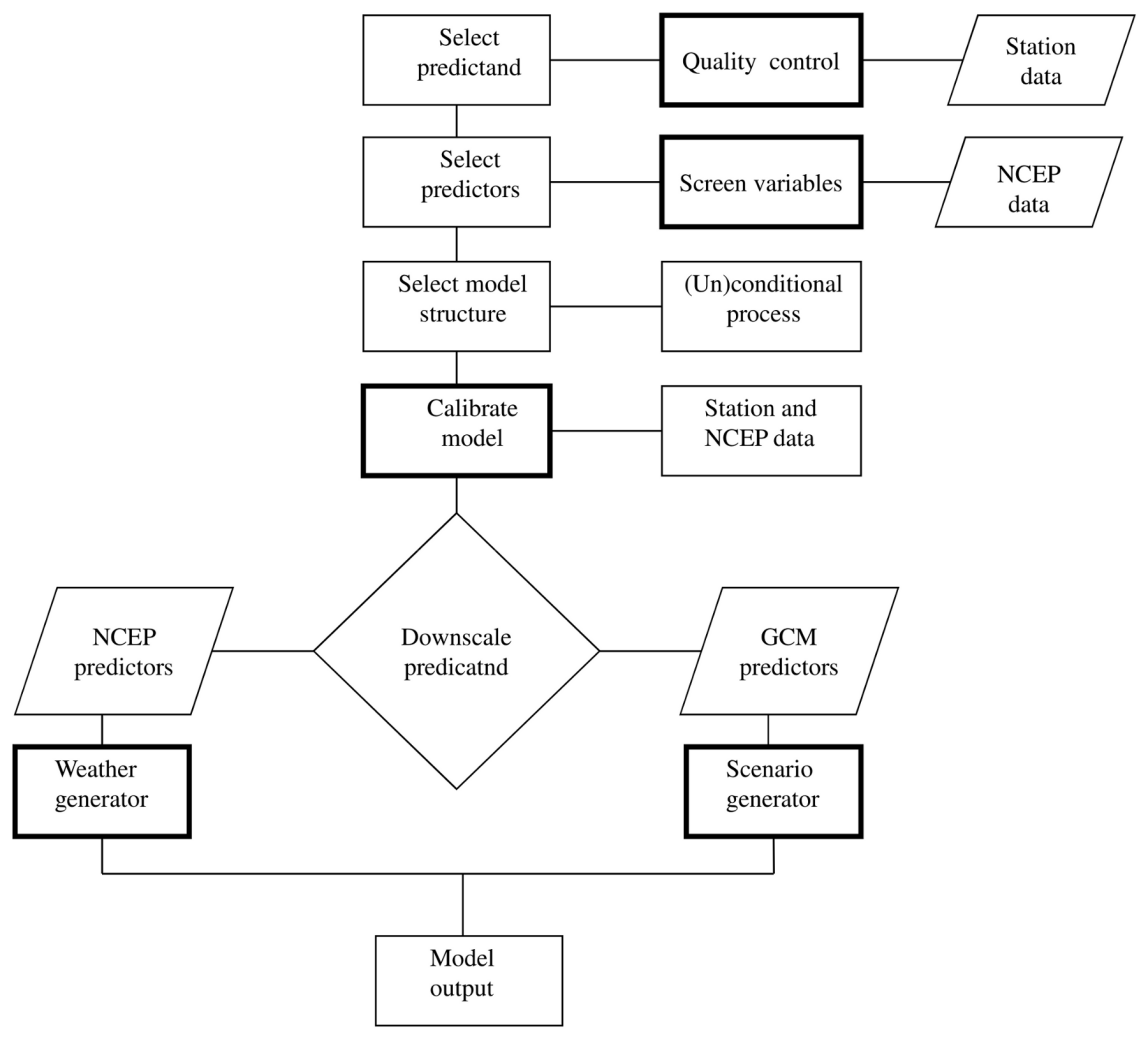
SDSM downscaling procedure (modified from Wilby and Dawson [33]).

Global Circulation Model (GCM) derived scenarios of climate change were used for predicting the future climates of the study area as they conform to criteria proposed by the Intergovernmental Panel on Climate Change (IPCC) [29]. The GCMs data are, however, too coarse in resolution to apply directly for impact assessment [30]. Thus, Statistical Down-Scaling Model (SDSM) was used to bridge this resolution gap. SDSM develops statistical relationships, based on multiple linear regression techniques, between large-scale (predictors) and local (predictand) climate [31–33]. The downscaling of GCMs data using SDSM was done following the procedures suggested by Wilby and Dawson [33]. The quality control option in SDSM was used for checking missing data and outliers. The screen variable option (a procedure in SDSM) was used to choose the appropriate downscaling predictor variables for model calibration. An unconditional process was selected for maximum and minimum temperature downscaling since the predictor-predictand process in temperature downscaling is not regulated by an intermediate process (cf. [33]). In unconditional models a direct link is assumed between the predictors and predictand (e.g., there is no intermediate process between maximum temperature (predictand) and near surface specific humidity (predictor)). While in conditional models, there is an intermediate process between predictors and predictand (e.g. precipitation amounts depend on the occurrence of wet-days, which in turn depend on regional-scale predictors such as humidity and atmospheric pressure). Thus for precipitation downscaling a conditional process was assumed. The significance level which tests the significance of predictor-predictand correlation was set to P-value <0.05. The model calibration process in SDSM was used to construct downscaled data based on multiple regression equations given daily weather data (predictand) and regional scale atmospheric variables (predictor). The ordinary least squares optimization technique was used to calibrate the model. The calibrated model was used to generate synthetic daily weather series using the observed atmospheric predictor variables and regression model weights. Validation in SDSM is evaluating the agreement between the generated weather series and an independent observed weather data excluded from model calibration process. The procedures of SDSM downscaling is provided in Figure 2. 

Large-scale predictor variable information was obtained from the National Center for Environmental Prediction (NCEP_1961-2001) reanalysis data set for the calibration and validation, and HadCM3 (Hadley centre Climate Model 3) GCM (H3A2a_1961-2099 and H3B2a_1961-2099) data for the baseline and climate scenario periods [34]. The HadCM3 GCM output was chosen as representative for the experimental area. When comparing output from different GCMs such as ECHAM4 (European Centre Hamburg Model 4), HadCM3, GFDL (Geophysical Fluid Dynamics Laboratory’s Rhomboidal 30 truncation), and CCSR (Center for Climate System Research), they all predict increase in precipitation for the Upper Blue Nile Basin during, e.g., the 2040-69 period [35]. Thus, it can be said that the HadCM3 is consistent with most other GCM models. The spatial variation for precipitation of GCM output, however, varies depending on model. The HadCM3 GCM predictions for the Upper Blue Nile Basin regarding precipitation amount and spatial variability are not extreme as compared to other GCMs but rather moderate [35] and thus, can be assumed to be representative for several GCMs. Future precipitation and temperature scenarios were generated using A2 (medium-high) and B2 (medium-low) emission scenarios of the IPCC Special Report on Emission Scenarios for the period 1961-2100 [35,36]. The HadCM3 predictor variables for the A2a and B2a experiments (the “*a*” in A2*a* and B2*a* refers the ensemble member in the HadCM3 A2 and B2 experiments [37]) were obtained on a grid by grid box basis for the study area from the Environment Canada website [34]. The predictor data represented a resolution of 2.5° latitude by 3.75° longitude. The predictors of the NCEP and HadCM3 GCM experiment with descriptions are presented in Table 1.

**Table 1:** Daily predictor variable held in the grid box data archive.

**Variable**	**Description**
temp	Mean temperature at 2 m
mslp	Mean sea level pressure
p500	500 hPa geopotential height
p850	850 hPa geopotentail height
rhum	Near surface relative humidity
r500	Reative humidity at 500 hPa height
r850	Relative humidity at 850 hPa height
shum	Near surface specific humidity
s500	Specific humidity at 500 hPa height
s850	Specific humidity at 850 hPa height
**Derived variable**	**Description**
**_f	Geostrophic air flow velocity
**_z	Vorticity
**_u	Zonal velocity component
**_v	Meridional velocity component
**zh	Divergence
**th	Wind direction

The derived variables have been derived using the geostrophic approximation [33].

** refers to different atmospheric levels: the surface (p_), 850 hPa height (p8) and 500 hPa height (p5)

The predictand variables used were precipitation and maximum and minimum temperature at Dangila station (Figure 1), and the data was obtained from the Ethiopian National Meteorological Services Agency [38]. This meteorological station was used for downscaling since it has long-term and high-quality data. All stations in the drainage basin are located within the same grid box. Consequently, the climate projection results from this station were assumed to represent other stations in the drainage basin. 

The NCEP reanalysis data that were used to calibrate and validate the SDSM model covered the period 1960-2001. The observed data from the Ethiopian National Meteorological Services Agency were for the period 1990-2001; the data from 1990-1997 and 1998-2001 were used for model calibration and validation, respectively. A monthly temporal resolution of the downscaled data was used to derive model parameters. An ensemble size of 20 values was generated, and the mean of ensemble members was used for the model validation process even though individual ensemble members were equally plausible. 

The calibrated model was used to generate ensemble members of synthetic daily weather series giving daily atmospheric predictor variables from the HadCM3 A2a and B2a experiment. The scenario generation produced 20 ensemble members of synthetic weather data for 139 years (1961-2099), and the mean of the ensemble members was calculated and used for impact assessment. It was adequate to consider the mean of the ensemble members since the aim was to reveal general trends in climate change. The generated scenario was divided into three time windows of 30 years of data centered at 2025 (2010-2039), 2055 (2040-2069) and 2085 (2070-2100) henceforth called 2020s, 2050s and 2080s, respectively. 

The Soil and Water Assessment Tool (SWAT) was applied for the Lake Tana Basin to assess the impact of climate change on the Gilgel Abay River and the Lake Tana. SWAT is a physically based model developed to predict the impact of land management practices on water, sediment, and agricultural chemical yields in watersheds with varying soil, land use, and management conditions [39]. SWAT has previously been applied in the highlands of Ethiopia and has given satisfactory results [28,40–42]. It simulates the hydrological cycle, vegetation growth, and nutrient cycling with a daily time step by disaggregating a river basin into sub-basins and hydrologic response units (HRU). HRUs are lumped land areas within the sub-basin that are comprised of unique land cover, soil, and management combinations. This allows the model to reflect differences in evapotranspiration and other hydrologic conditions for different land cover and soil types [39]. SWAT has different options to calculate the hydrological components in a watershed. In this study, Hargreaves’s method was used for the determination of potential evapotranspiration. Many studies (e.g., [40,42]) have applied Hargreaves’s method in the Lake Tana Basin with satisfactory results. Surface runoff was estimated using the Soil Conservation Service’s curve number method, which is a nonlinear function of precipitation and retention coefficient [43]. The surface runoff was estimated separately for each HRU and routed to obtain the total runoff for the watershed. Variable storage routing method was selected for routing the flow of water in the channels. 

#### Spatial data

A digital elevation model (DEM) was used to delineate the sub-watersheds in the ArcSWAT interface [44]. The ArcSWAT is an ArcGIS extension for the SWAT graphical user interface. The DEM data was obtained from the CGIAR Consortium for Spatial Information (CGIAR-CSI) website [45], and has a resolution of 90 m by 90 m. The stream network dataset was superimposed onto the DEM to define the location of the streams. The land use and soil data defined the HRUs. The stream network, land use/land cover, and soil maps of the study area were obtained from the Ethiopian Ministry of Water Resources [46]. The soil physical and chemical properties required by SWAT were derived from the digital soil map of the world CD-ROM Africa map sheet [47].

#### Hydrometeorological data

The climatic data that were used for SWAT model setup were also obtained from the Ethiopian National Meteorological Services Agency [38]. These data consist of precipitation and maximum and minimum temperature from nine stations located within and around the study watershed (Figure 1). The SWAT in-built weather generator was used to fill in missing data, and to generate other climatic inputs such as solar radiation, relative humidity, and wind speed. The daily river flow at the Gilgel Abay gauging station (Figure 1), which was obtained from the Ethiopian Ministry of Water Resources [46], was used for SWAT calibration, validation, and climate change impact analysis. Calibration of SWAT was performed by adjusting model parameters within their physical acceptable intervals to achieve a reasonable agreement between observed and simulated stream flows (cf. [48]). Validation of SWAT, on the other hand, was the process of testing the performance of the calibrated model (i.e. the agreement between observed and simulated stream flows) without changing parameter values that were set during the calibration, when simulating the response for a period other than the calibration period [48]. 

SWAT is a complex model with many parameters making calibration complex. Hence, sensitivity analysis was performed to delimit the number of parameters which affected the fit between simulated and observed data. A combination of Latin Hypercube Sampling and One-Factor-At-a-Time sensitivity analysis methods were used [49]. The concept of Latin-Hypercube simulation is based on Monte Carlo simulation to allow for a robust analysis but a stratified sampling approach for efficient estimation of output statistics while the One-Factor-At-a-Time is an integration of a local to a global sensitivity method [49]. 

The calibration of the model was made for a period of six years from 1995-2000. SWAT has two calibration tools: manual calibration helper and auto-calibration. Initially, manual calibration was used and when the model objective functions reached a satisfactory level (i.e., Nash-Sutcliff efficiency (E_NS_) [50] of above 0.5), the auto calibration process continued. The manual calibration was done by partitioning stream-flow into surface runoff and base-flow. The surface runoff was calibrated by adjusting sensitive parameters which affect surface runoff (e.g., CN2 – Initial SCS runoff curve number for moisture condition II, Ch_N2 – Manning’s ‘’n’’ value for the main channel, and Esco – Soil evaporation compensation factor). Calibration of surface runoff was performed until a satisfactory objective function was achieved (i.e., E_NS_ >0.5). Thereby, calibration of base-flow parameters followed by adjusting the sensitive parameters which affects groundwater contribution (e.g., GW_REVAP – Groundwater ‘’revap’’ coefficient, and GWQMN – threshold depth of water in the shallow aquifer required for return flow to occur). Similarly, these parameters were adjusted until the E_NS_ value was above 0.5. After every adjustment of base-flow parameters, the surface runoff was checked since the adjustment of base-flow parameters might affect the surface runoff simulation. Once the water balance was calibrated, temporal flow calibration was performed at each step by adjusting parameters which affect the shape of the hydrograph (e.g., Ch_K – effective hydraulic conductivity in main channel alluvium, alpha_BF – baseflow alpha factor, Surlag – surface runoff lag coefficient, and GW_DELAY – groundwater delay time). The manually calibrated parameter values were set as initial values for the auto-calibration process. Finally, the model was validated with a stream flow data from 2001-2004.

The performance of SWAT was evaluated using statistical measures to determine the quality and reliability of predictions compared to observed values. The Nash-Sutcliffe simulation efficiency (E_NS_) was the goodness of fit measure used to evaluate model prediction. The E_NS_ is a normalized statistic that determines the relative magnitude of the residual variance compared to the measured data variance [50]. E_NS_ can range from -∞ to 1. An E_NS_ value of 1 corresponds to a perfect match of observed stream flow to the simulated stream flow. An E_NS_ between 0 and 1 are considered as acceptable levels of performance, whereas an E_NS_ ≤ 0 suggests the observed mean is a better predictor than the model. 

SWAT has the capability to simulate the impact of climate change through adjustments in the climatic inputs that is read into the model. In this study changes in precipitation and temperature were applied to analyze the effect of climate change on the river flow. The adjustment terms from month to month climate variables were derived from the SDSM climate change downscaling results. 

## Results

### Climate projection

**Figure 3:**
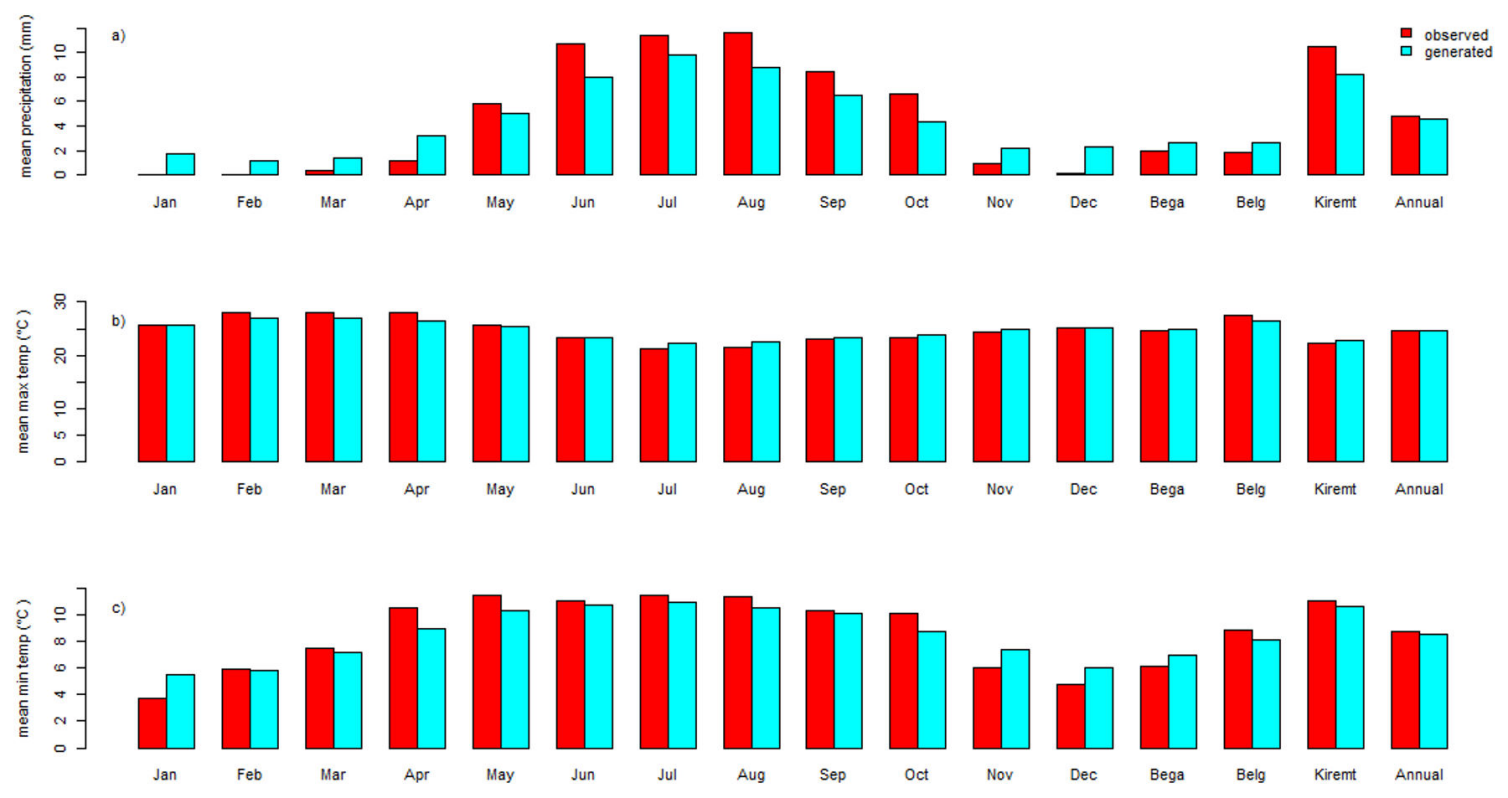
Comparision between observed and generated mean daily precipitation and maximum and minimum temperature in the time step for the Dangila station. a) precipitation (mm), b) maximum temperature (°C), and c) minimum temperature (°C). Bega season = October–January, Belg season = February–May, and Kiremit season = June - September.

The SDSM model resulted in satisfactory multiple regression equation parameters for maximum and minimum temperature (Table 2). Thus, it may be inferred that future projections may also be well replicated [33]. The precipitation downscaling was, however, characterized by not as good calibration and verification (Table 2). Rainfall predictions, however, have a larger degree of uncertainty than those for temperature since rainfall is highly variable in space and the relatively coarse GCM models cannot adequately capture this variability [33,51]. However, a graphical comparison between observed average long term mean monthly precipitation, and maximum and minimum temperature with corresponding simulations showed that the results of the SDSM model replicated the basic pattern of observations (Figure 3).

**Table 2:** R^2^ for calibration and validation of SDSM downscaling of precipitation, and maximum and minimum temperature at the Dangila station.

	Precipitation	Maximum temperature	Minimum temperature
Calibration	0.42	0.49	0.47
Validation	0.31	0.52	0.46

**Figure 4:**
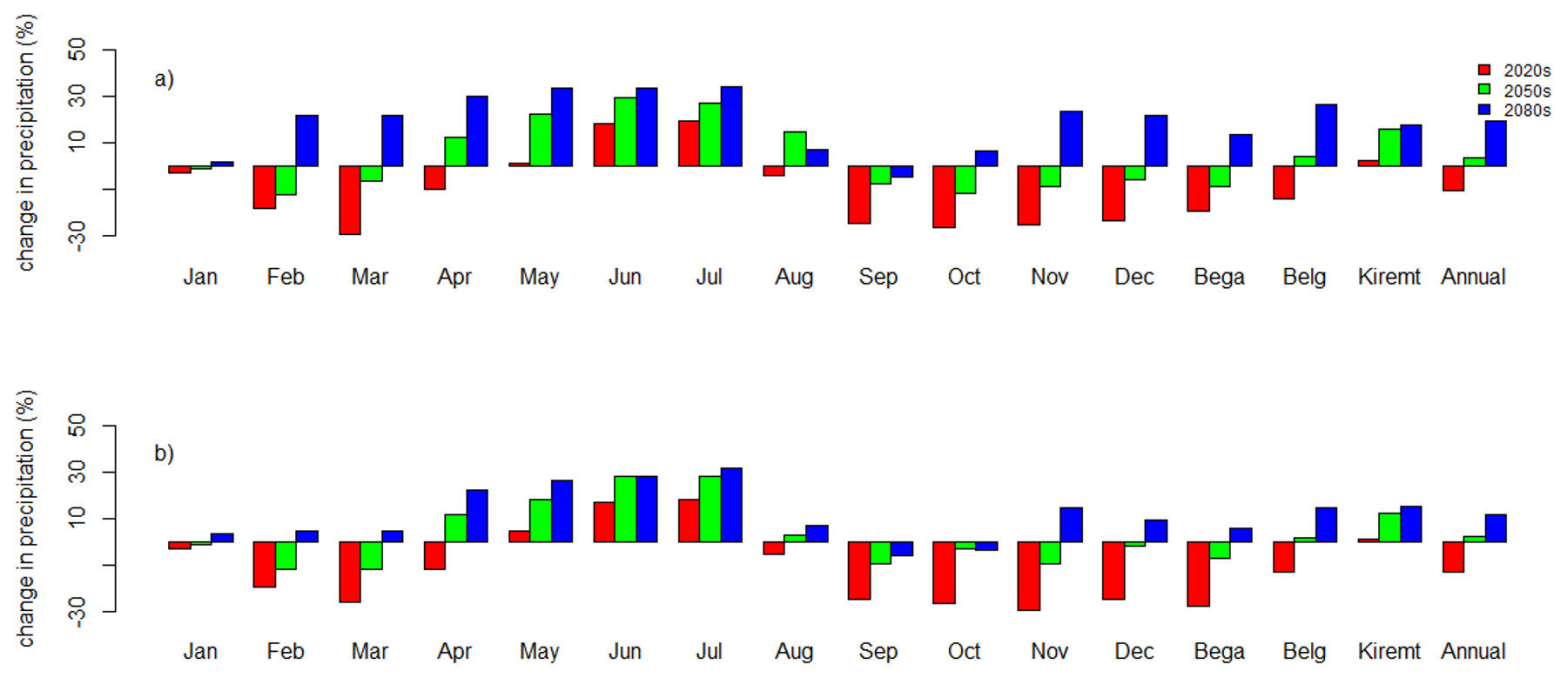
Percentage change in monthly, seasonal, and annual precipitation for the period 2010-2099 as compared to the baseline period (1990-2001) at Dangila station. a) A2a scenario and b) B2a Scenario. Bega season = October–January, Belg season = February–May, and Kiremit season = June -September.

Results showed a general decrease in annual mean precipitation for the 2020s and an increase for the 2050s and 2080s (Figure 4). As shown in Figure 4, in the 2020s there may be a decrease in mean monthly precipitation for all months except May, June, and July for both scenarios (A2a and B2a). For the 2020s, the A2a and B2a scenarios displayed both a mean monthly precipitation decrease by -29% and -30%, respectively. In the 2020s, mean monthly precipitation increase reached +19% for the A2a scenario and +18% for the B2a scenario. The annual mean precipitation in the 2020s may decrease by -10% and -13% for the A2a scenario and B2a scenario, respectively. For the 2050s, there may be an early occurrence and early end of precipitation as compared with the baseline period. This is reflected by an increase of mean monthly precipitation in April and a decrease in September. The overall effect in the 2050s was a small increase of annual mean precipitation by +4% for the A2a case and +2% for the B2a case. For the 2050s, an increase in mean monthly precipitation was indicated by +29% for the A2a scenario and +28% for the B2a scenario. For the 2050s, a decrease in mean monthly precipitation was indicated corresponding to -12% for the A2a scenario and -14% for the B2a scenario. For the 2080s, results indicate an increase in mean monthly precipitation during all months except in September for the A2a scenario and September and October for the B2a scenario. The increase in mean monthly precipitation was +34% for the A2a scenario and +32% for the B2a scenario. The A2a and B2a scenarios showed an increase in annual mean precipitation by +19% and +12%, respectively. 

The above results would mean a generally increasing precipitation during the Kiremit (wet season = June–September) for the long-term future (Figure 4). Results also indicate a corresponding increase in precipitation for the Belg (less rainy season = February–May) for 2050s and 2080s. The Kiremit and Belg are the cropping seasons in Ethiopia. This gives an insight into the possible impact of climate change on agriculture in the study area. 

**Figure 5:**
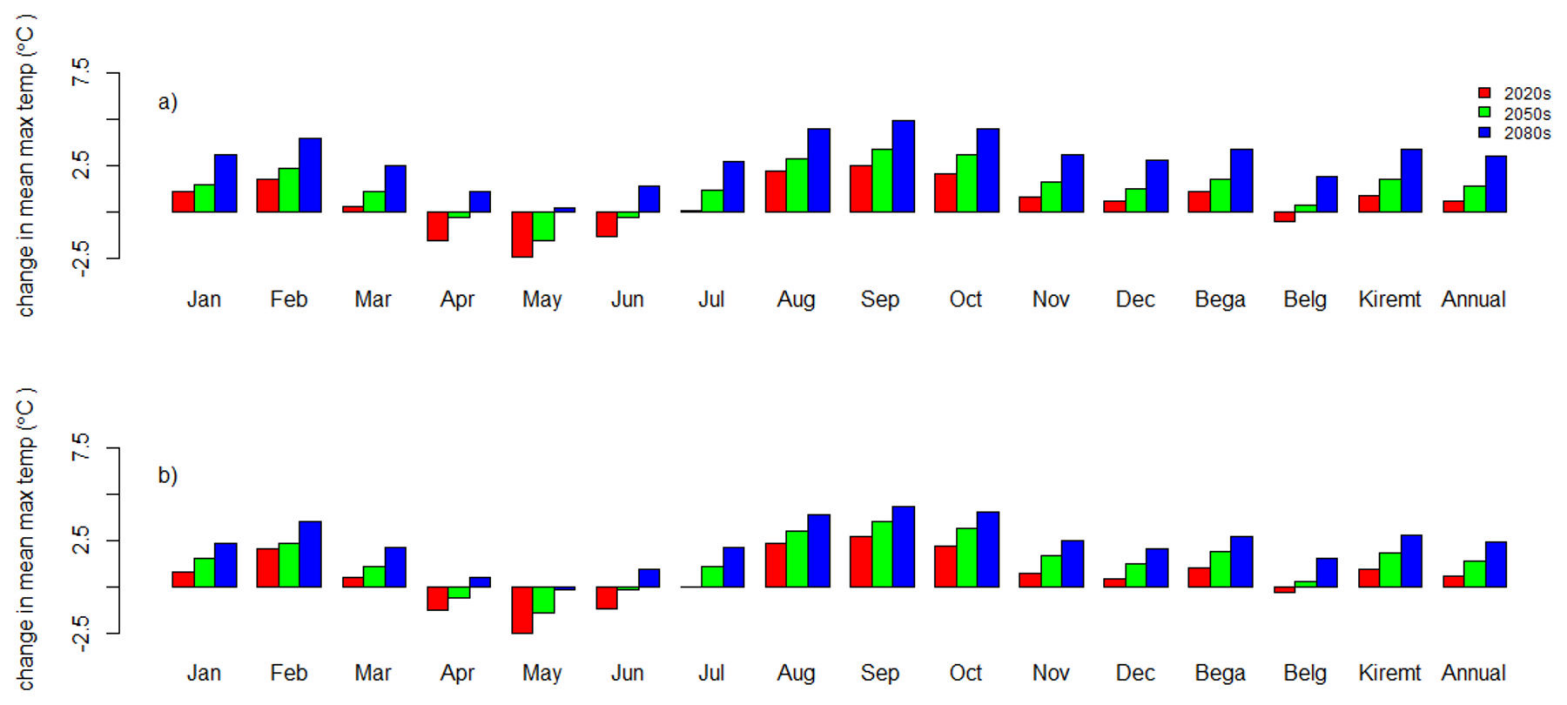
Change in monthly, seasonal and annual mean maximum temperature for the period 2010-2099 as compared to the baseline period (1990-2001) at Dangila station. a) A2a scenario and b) B2a scenario. Bega season = October–January, Belg season = February–May, and Kiremit season = June–September.

The maximum temperature scenario showed that there may be an increase in mean monthly maximum temperature for all months except for April, May, and June for the 2020s and 2050s (Figure 5). However, mean monthly maximum temperature increased for all months in the 2080s (except for May in B2a scenario). The change in mean monthly maximum temperature ranged between -2.4 °C in May (2020s) and +5 °C in September (2080s) for the A2a scenario, and between -2.5 °C in May (2020s) and +4.3 °C in September (2080s) for the B2a scenario. Seasonally, a pronounced increase in mean maximum temperature is indicated during the Bega (dry season = October–January) and Kiremit. The mean monthly, seasonal, and annual change in daily maximum temperature from the baseline period data are shown in Figure 5.

The overall results (2010-2099) for annual mean maximum temperature showed an increasing trend for both scenarios (A2a and B2a). The mean maximum temperature increase is +0.52 ^o^C/decade and +0.34 ^o^C/decade for A2a and B2a scenarios, respectively. 

**Figure 6:**
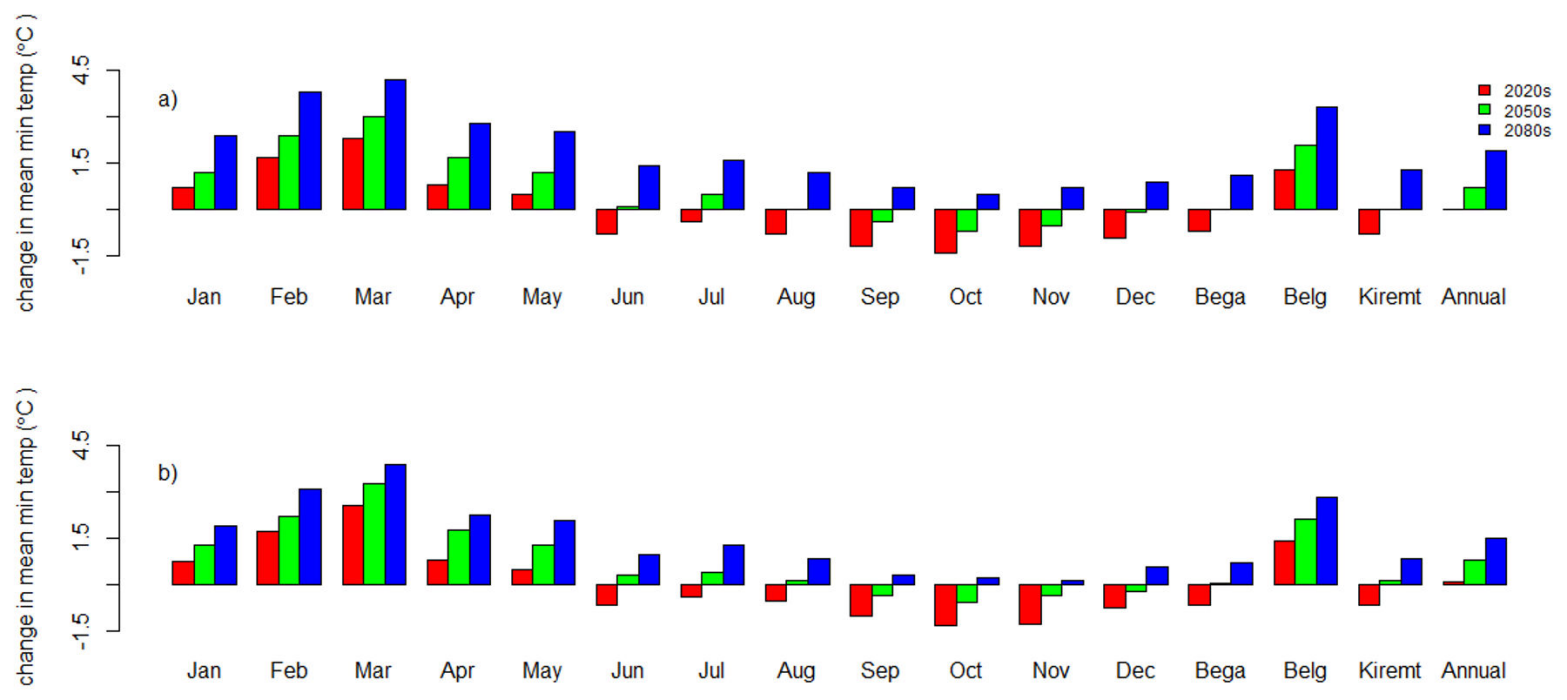
Change in monthly, seasonal, and annual mean minimum temperature for the period 2010-2099 as compared to the baseline period (1990-2001) at the Dangila station. a) A2a scenario, b) B2a scenario. Bega season = October–January, Belg season = February–May, and Kiremit season = June–September.

The annual mean minimum temperature trend increases for both scenarios (Figure 6). The annual mean minimum temperature change for 2020s may be negligible. However, for the 2050s all months except September, October, November, and December display an increase in mean monthly minimum temperature for both scenarios. For this period the effect is an increase in mean minimum temperature during the Belg season. For the 2080s, there may be an increase in mean monthly minimum temperature for all months but the increase will mainly be significant for the Belg months. The change in mean monthly minimum temperature ranges between -1.4 °C in October for the 2020s and +4.2 °C in March for the 2080s for the A2a scenario, and -1.3 °C in October for the 2020s and +3.8 °C in March for the 2080s for the B2a scenario. 

The long term trend analysis (1990-2099) showed that the annual mean minimum temperature may increase by +0.43 ^o^C/decade and +0.27 ^o^C/decade for the A2a and B2a scenarios, respectively. 

### Hydrological modeling

The watershed delineation and HRU definition for the Lake Tana Basin resulted in 34 sub-basins and 189 HRUs with a total watershed area of 14,952 km^2^. Delineation of the watershed gave minimum, maximum, and mean elevation in the basin as 1,759, 4,109, and 2,025 m amsl, respectively. 

The analysis included testing the degree of sensitivity of 26 flow parameters and their parameter bound. Alpha_Bf (baseflow alpha factor) and Gw_Delay (groundwater delay time) were the most sensitive parameters that affected the base-flow contribution while CN2 (curve number for moisture condition-II), Esco (soil evaporation compensation factor), Ch_N2 (Manning’s ‘’n’’ value for the main channel), and Surlag (surface runoff lag coefficient) were among the most sensitive parameters for surface runoff. The eight most sensitive parameters, their ranking, and description are shown in Table 3.

**Table 3:** Parameter sensitivity ranking and final auto-calibration results.

Rank	Parameter	Description	Range	Auto-calibrated result
1	Alpha_Bf	Baseflow alpha factor	0-1	0.4
2	CN2	Initial SCS runoff curve number for moisture condition II	-25% -+25%	-5%
3	Ch_N2	Manning’s “n” value for the main channel	0.01-0.3	0.059
4	Ch_K2	Effective hydraulic conductivity in main channel alluvium	0.01-150	122.86
5	Surlag	Surface runoff lag coefficient	0-10	4
6	Esco	Soil evaporation compensation factor	0-1	0.95
7	Gw_Delay	Groundwater delay time	0-500	30.7
8	GW_REVAP	Groundwater “revap” coefficient	0.02-0.2	0.18

The manual calibration resulted in a reasonable agreement between daily observed and simulated streamflow (E_NS_ = 0.54). The auto-calibration improved the E_NS_ values to 0.74. In Santhi et al. [52],, the authors suggested that efficiency values greater than or equal to 0.50 are considered adequate for SWAT model application. Table 4 also exhibited good agreement between simulated and measured data. 

**Table 4:** Calibration and validation statistics for measured and simulated flows at Gilgel Abay River flow gauge station.

	Total flow (m^3^/s)		Average flow (m^3^/s)		
Period	Observed	Simulated	Observed	Simulated	% error
Calibration (1995-2000)	129077	124757	58.88	56.91	3.34
Validation (2001-2005)	71196	70657	48.73	48.36	0.75

**Figure 7:**
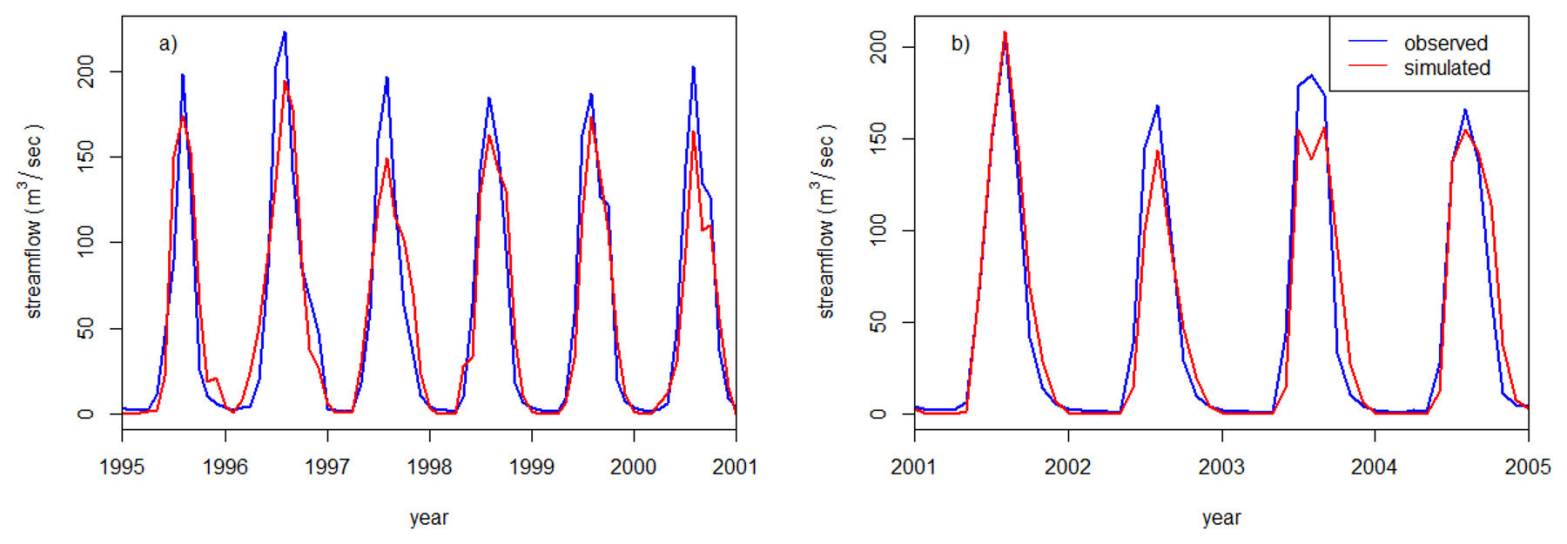
Hydrograph between mean monthly observed and simulated stream flows at the Gilgel Abay gauging station. a) calibration period, and b) validation period.

The hydrographs (Figure 7a) for mean monthly observed and simulated stream flows after calibration showed a reasonable agreement. Validation of the model confirmed the model’s strong predictive capability through E_NS_ values of 0.78 (Figure 7b). Other statistical measures (Table 4) also proved SWAT’s strong performance in the basin. 

From the calibration and validation results, it may be deduced that the model represents the hydrological characteristics of the watershed and can be used for further analysis. 

### Climate change impact

**Figure 8:**
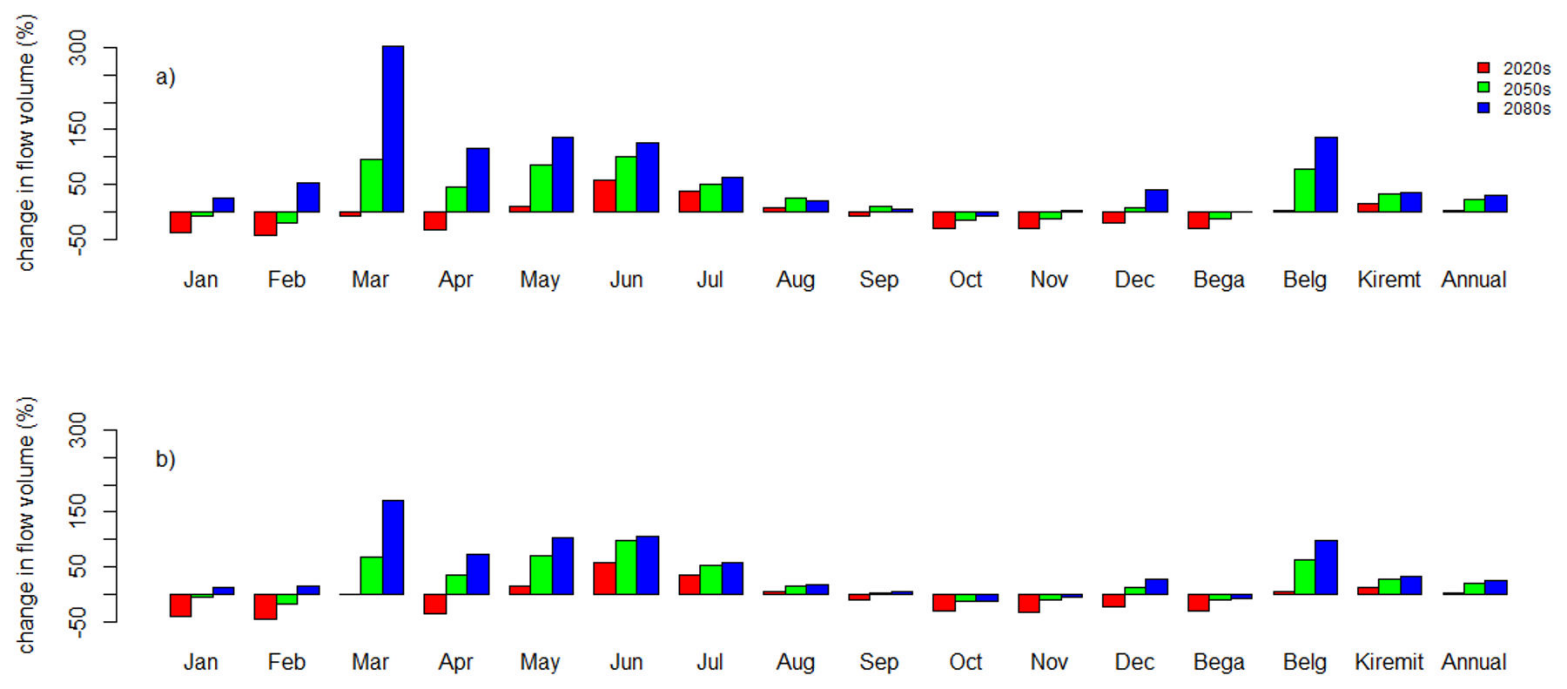
Percentage change in mean monthly, seasonal, and annual flow volume for the period 2010-2099 as compared to the baseline period (1990-2001) at the Gilgel Abay gauging station. a) A2a scenario and b) B2a scenario. Bega season = October–January, Belg season = February–May, and Kiremit season = June–September.

The impact of climate change on stream flow was predicted based on conditional temperature and rainfall changes on a monthly, seasonal, and annual basis. The effect of climate change on low flow was also analyzed. The analysis was done taking the 1990-2001 river flow as baseline flow against which the future flows for the 2020s, 2050s, and 2080s were compared. The percentage change in mean monthly flow volume in both scenarios and for the periods 2020s, 2050s, and 2080s is presented in Figure 8. 

For the 2020s, the mean monthly flow volume shows a decrease for all months except May-August in the A2a scenario. For this period a decrease of -43% and an increase of +58% in mean monthly flow volume are indicated. Increase in mean monthly flow volume is observed for months which show a corresponding increase in mean monthly precipitation. However, August displays a decrease in mean monthly precipitation by -4% but an increase in mean monthly flow volume by about +7%. The increase in mean monthly flow volume in August is mainly due to catchment and groundwater lag time effects. For the 2020s in the B2a scenario, the same effect as in the A2a scenario of 2020s is observed. 

For the 2050s, increase in precipitation is reflected in an increase in flow volume and vice versa. March and September however, displayed a decrease in mean monthly precipitation but an increase in mean monthly flow volume. For September this is attributed to the effect of the increase in precipitation during the previous months. March’s exceptional increase in mean monthly flow volume is more difficult to explain. In the 2050s, the mean monthly flow volume is indicated to increase by +100% and decrease to -20% for the A2a scenario and increase to +97% and decrease to -19% for the B2a scenario.

For the 2080s and the A2a scenario, an increase in mean monthly flow volume for all months except October is indicated. The increase in mean monthly flow volume may reach +135%, but a decrease in precipitation in September by -5% may decrease the mean monthly flow volume by -7% in October. For the 2080s B2a scenario, the pattern of mean monthly flow volume change is more or less the same as the A2a scenario and the increase in mean monthly flow volume is +106%. But a decrease in mean monthly precipitation in September and October by -6% and -4% respectively, results in a decreasing mean monthly flow volume in October and November by -13% and -5%, respectively. 

Results indicate an increase in annual mean flow volume for the 90-year prediction horizon (Figure 8). The Belg season shows the larger share in increased flow volume. The indicated increase is +136% for the 2080s in the A2a scenario and +99% for the 2080s in the B2a scenario. The Kiremit season also shows an increase in flow volume; the increase ranges from +15% to +36% for the A2a scenario and +12% to +32% for the B2a scenario. However, both scenarios indicate that there might be a decrease in flow volume during the Bega season. The decrease is about -30% for both A2a and B2a scenarios. 

**Figure 9:**
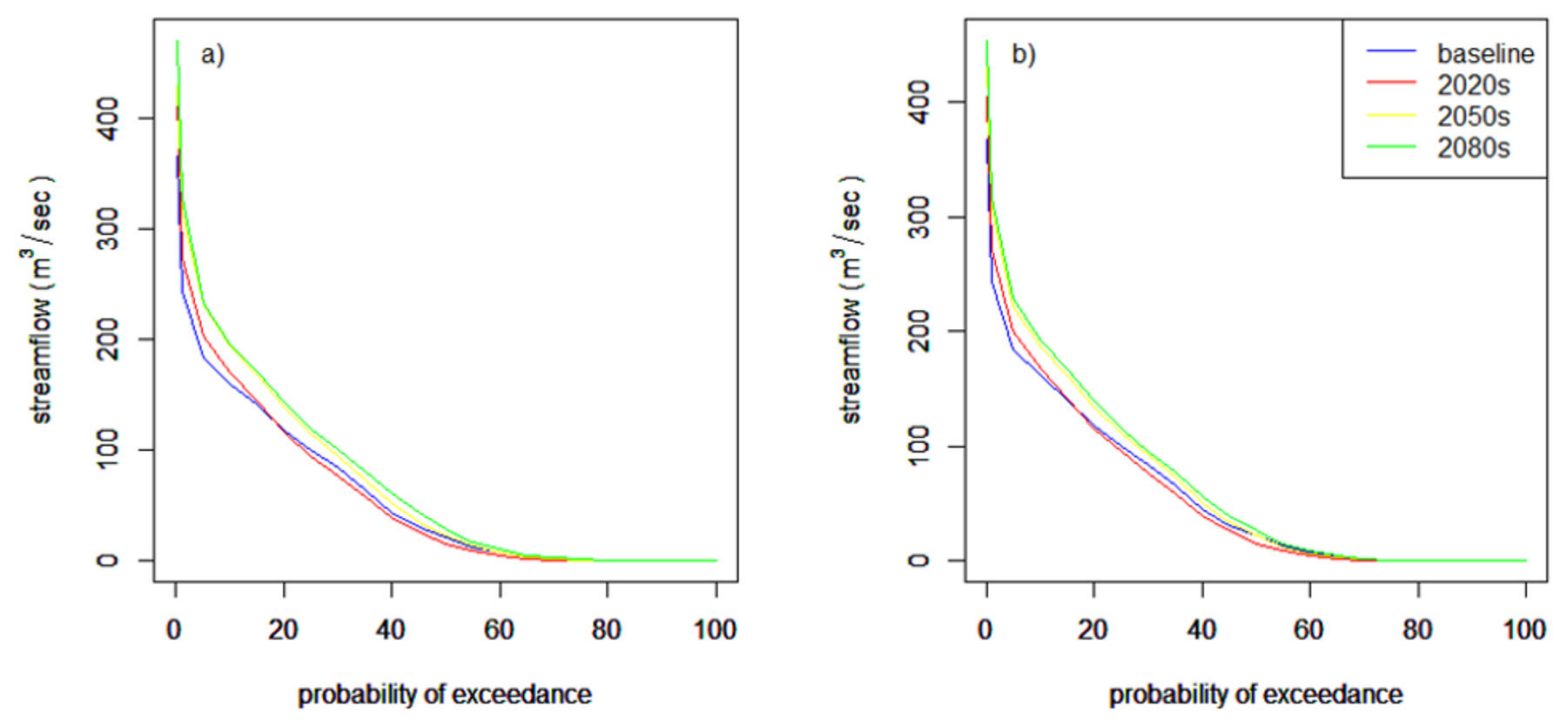
Flow duration curve to analyze the low flow at the Gilgel Abay gauging station. a) A2a scenario and b) B2a scenario.

Climate change will affect both high and low flows owing to variability in precipitation and temperature. Analyzing low flow statistics is important for water quality and aquatic habitat needs. Hydrologic regimes are often quantified using statistics of flow duration curves (*cf.* [35]). In this study a 95% flow exceedance probability was considered to characterize low flow conditions. It was found, however, that there is no major effect for low flows at this probability of exceedance. The effect is visible at 70% exceedance probability indicating that in the 2020s and 2050s the low flow may decrease but increase in the 2080s for both scenarios. Table 5 and Figure 9 show low flow statistics at 70% exceedance probability and corresponding flow duration curves, respectively.

**Table 5:** Low flow statistics for Gilgel Abay River flow for A2a and B2a scenarios at three time windows.

	Periods			
Scenarios	Baseline^*^	2020s	2050s	2080s
A2a	1.89	0.79	1.61	2.50
B2a	1.89	0.77	1.35	1.98

* baseline period is not under any of the scenarios

## Discussion and Conclusion

We predicted the conditional impact of rainfall and temperature changes on the hydrology of the Lake Tana Basin using the HadCM3 GCM A2a and B2a climatic scenarios for the 2010-2100 period. We applied the SDSM statistical downscaling tool to evaluate the GCM outputs. The SWAT model was used to study the consequences of climate change on the hydrology of Lake Tana Basin. We believe that results presented in this study are representative for a majority of GCM output and that therefore our results are plausible estimates of future effects of climate change. Our temperature projection results (an increase in mean monthly temperature up to +2.5 °C in the 2020s, +3.1 °C for the 2050s, +5 °C for the 2080s) are consistent with results reported by several other previous studies [10,19]. Even so, the SDSM precipitation weather generation could not satisfactorily replicate the observed precipitation due its inherent high variability in space [51]. Precipitation prediction for the Blue Nile is a difficult task with some climatic models projecting more, and other less precipitation [10,19,53,54]. The majority of GCMs, however, indicate a precipitation increase over the Upper Blue Nile Basin in a 50-100 year perspective. In line with this, our simulated precipitation results matched observed precipitation in a satisfactory way (Figure 3a). This suggests that SDSM may perform well in simulating the future climatic condition of the study area. As in any type of modeling study the results have to be judged against uncertainties. Even if we cannot quantify these uncertainties in this study it is well known that uncertainty increases along the sequence temperature-precipitation-runoff. The uncertainty also increases with smaller time and spatial scale. Consequently, results have to be viewed in this perspective. On the other hand, similarity in results with other studies using other approaches corroborates results. In any case, percentage changes’ of different hydrometeorological quantities as in this study should not be seen as facts but instead as an indication of possible future outcomes with a high degree of uncertainty.

In view of the above, the SDSM downscaling indicates an annual mean precipitation decrease for the 2020s by -10% (A2a scenario) and -13% (B2a scenario). The annual mean precipitation will increase by 3.8% and 2.2% for 2050s in the A2a and B2a scenarios. For the 2080s the annual mean precipitation may increase with +19.3% (A2a scenario) and 12% (B2a scenario). Seasonally, precipitation may increase in the Kiremit in the 90-year horizon as well as in the Belg after 2040. Abdo et al. [24] found that rainfall in the Kiremit season may decrease for the A2a scenario and the forthcoming century, a decrease in the 2020s, and a slight increase in the 2050s and 2080s for the B2 scenarios. A multimodal average precipitation projection for the entire Blue Nile basin by Beyene et al. [19] for the Kiremit showed an increase in precipitation for the 2020s and 2080s but a decrease in the 2050s. Our and partly Abdo et al.’s [24] and Beyene et al.’s [19] results’, indicate that rainfall may increase during the Kiremit season. As Kiremit and Belg are the cropping seasons in Ethiopia, climate change may have positive implications for the rainfed agricultural sector even if the increase in the maximum and minimum temperature has a contradictory effect by increasing evapotranspiration. 

The runoff is expected to change according to temperature and precipitation changes. The coming decades may thus bring less runoff (indicated by the simulated -46% decrease in mean monthly flow volume in the 2020s). However, the mid and later part of the coming century may bring increase in runoff (indicated increase up to +135% in mean monthly flow volume during the 2080s). There may be a significant increase in flow volume during the Belg season (indications of up to +136%) and an increase in flow volume during the Kiremit season (indications of up to +36%). Overall there may be an annual increase in flow volume in the Gilgel Abay river flow due to climate change. Taye et al. [23] showed that the mean annual flow change at the Lake Tana outlet may range from -81% to +75%. On the other hand, it has been found a dominant declining annual streamflow for the Gilgel Abay river using nine GCMs (other than HaDCM3) [54]. This owing to that the GCMs they applied showed a precipitation decrease. Similarly, Abdo et al. [24] observed a decrease in runoff for the coming century except in the 2050s. We may conclude that the difference in results is mainly a result from the different GCM results. Based on our results, the increase in Belg season flow would have paramount importance for small scale irrigation activities practiced by local farmers. As the Gilgel Abay is the largest tributary river feeding in to Lake Tana, any change in river flow is likely to affect the lake. Besides, as the basin is small, it is assumed that the impact of climate change will be more or less the same in other tributary rivers. Hence, it can be concluded that climate change overall may result in an increase in flow volume into Lake Tana based on HadCM3 rainfall and temperature projections. This may have positive as well as negative implications for the socio-economic conditions of the region. The increase in flow will help to harness a significant amount of water for the ongoing dam projects in the Gilgel Abay River basin. However, it may also aggravate the recurrent flooding problems in the area surrounding Lake Tana.

The cascade of models used in this study showed a satisfactory performance. Even so, it is undeniable that there are uncertainties due to inherent assumptions in all used models [55]. In general, many of these uncertainties are related to the generation of regional climate information from the general climate change scenarios [56]. These include uncertainties regarding future emissions of greenhouse gases, differing responses of GCMs to the resulting concentrations of emissions, and uncertainties related to downscaling techniques [55,57]. Downscaled scenarios in this study were generated using only one GCM model experiment. Downscaled scenarios using other GCM models running the same experiment may likely produce different, but equally plausible results [58]. Especially precipitation changes simulated by GCMs in much of Africa involve considerable uncertainty [55,59,60] because of the inability of climate model predictions to account for the influence of land cover changes on future climate and the relatively poor representation in many models of some aspects of climate variability that are important for Africa (e.g., ENSO) [60]. For example there is not a clear agreement between different GCMs how the rainfall in the Sahel, the Guinean Coast and the southern Sahara will change [55,60–63]. However, there is a robust increase in rainfall in East Africa, with 18 of 21 models projecting an increase in the core of this region [55]. We observed a general increase in precipitation with HaDCM3 downscaling in the Lake Tana Basin. This suggests that increase in runoff could be one of the plausible scenarios. Uncertainties are also pertinent to the hydrologic modeling applied for the impact assessment [64]. These include input uncertainty, parameter uncertainty, and model structure uncertainty. Moreover, we assumed that the land cover will remain the same over the analysis period; however, the land cover will change due to natural and anthropogenic influences. Hence the results of this study should be considered as indicators of the future changes on climate and hydrology rather than actual estimations. However, results presented in this paper can still provide invaluable insight to decision makers on the degree of vulnerability of Lake Tana Basin to climate change, which is important to design appropriate adaptation and mitigation strategies. 

## References

[1] Bates B, Kundzewicz Z, Wu S, Palutikof J (2008) Climate Change and Water. Technical Paper of the Intergovernmental Panel on Climate Change. Geneva.

[2] Arnell NW (2004) Climate change and global water resources: SRES emissions and socio-economic scenarios. Glob Environ Chang 14: 31–52. doi:10.1016/j.gloenvcha.2003.10.006.

[3] Vörösmarty CJ, Douglas AG, Ravenga C (2005) Geospatial indicators of emerging water stress: an application to Africa. Ambio 34: 230–236. doi:10.1639/0044-7447(2005)034[0230:GIOEWS]2.0.CO;2. PubMed: 16042282. 16042282

[4] Ashton PJ (2002) Avoiding conflicts over Africa’s water resources. Ambio 31: 236–242. doi:10.1639/0044-7447(2002)031[0236:ACOASW]2.0.CO;2. PubMed: 12164134. 12164134 10.1579/0044-7447-31.3.236

[5] CSA (2007) Summary and statistical report of the 2007 population and housing census. Ethiopia: Addis Ababa.

[6] IFAD (2009) Federal Democratic Republic of Ethiopia: Country Program Evaluation.

[7] Bank World (2006) Country Water Resources Assistance Strategy Ethiopia: Managing Water Resources to Maximize Economic Growth. Washington.

[8] Fischer G, Shah M, Tubiello FN, van Velhuizen H (2005) Socio-economic and climate change impacts on agriculture: an integrated assessment, 1990-2080. Philos Trans R Soc Lond B Biol Sci 360: 2067–2083. doi:10.1098/rstb.2005.1744. PubMed: 16433094. 16433094 PMC1569572

[9] IPCC (2012) Summary for Policymakers. In: Managing the Risks of Extreme Events and Disasters to Advance Climate Change Adaptation. Field CB Barros V Stocker TF Qin D Dokken DJ Cambridge, UK, and New York, NY, USA: Cambridge University Press.

[10] Conway D, Schipper EL (2011) Adaptation to climate change in Africa: Challenges and opportunities identified from Ethiopia. Glob Environ Chang 21: 227–237. doi:10.1016/j.gloenvcha.2010.07.013.

[11] Müller C, Cramer W, Hare WL, Lotze-Campen H (2011) Climate change risks for African agriculture. Proc Natl Acad Sci U S A 108: 4313–4315. doi:10.1073/pnas.1015078108. PubMed: 21368199. 21368199 PMC3060257

[12] Conway D, Hulme M (1996) The impacts of climate variability and future climate change in the Nile basin on water resources in Egypt. Int J Water Resour Dev 12: 261–280. doi:10.1080/07900629650169.

[13] Gleick P (1991) The vulnerability of runoff in the Nile basin to climatic changes. Environ Prof 13: 66–73.

[14] Hailemariam K (1999) Impact of climate change on the water resources of Awash River Basin , Ethiopia. Clim Chang 12: 91–96.

[15] Kim U, Kaluarachchi J, Smakhtin U (2008) Generation of monthly precipitation under climate change for the Upper Blue Nile river basin. J Am Water Resour Assoc 44: 1–17. doi:10.1111/j.1752-1688.2007.00134.x.

[16] Legesse D, Vallet-Coulomb C, Gasse F (2003) Hydrological response of a catchment to climate and land use changes in Tropical Africa: case study South Central Ethiopia. J Hydrol 275: 67–85. doi:10.1016/S0022-1694(03)00019-2.

[17] Deressa TT, Hassan RM, Ringler C, Alemu T, Yesuf M (2009) Determinants of farmers’ choice of adaptation methods to climate change in the Nile Basin of Ethiopia. Glob Environ Chang 19: 248–255. doi:10.1016/j.gloenvcha.2009.01.002.

[18] Xu C (2000) Climate Change and hydrologic models : a review of existing gaps and recent research developments. Water Resour Manag 13: 369–382.

[19] Beyene T, Lettenmaier DP, Kabat P (2010) Hydrological impacts of climate change on the Nile River Basin: Implications of the 2007 IPCC scenarios. Clim Chang 100: 433–461. doi:10.1007/s10584-009-9693-0.

[20] Gibson CC, Ostrom E, Ahn T (2000) The concept of scale and the human dimensions of global change: a survey. Ecol Econ 32: 217–239. doi:10.1016/S0921-8009(99)00092-0.

[21] Wilbanks TJ, Kates RW (1999) Global change in local places: how scale matters. Clim Chang 43: 601–628. doi:10.1023/A:1005418924748.

[22] Tarekegne D, Tadege A (2006) Assessing the impact of climate change on the water resources of the Lake Tana basinusing the Watbal model. University of Pretoria.

[23] Taye M, Ntegeka V, Willems P (2011) Assessment of climate change impact on hydrological extremes in two source regions of the Nile River Basin. Hydrol Earth Syst Sci 15: 209–222. doi:10.5194/hess-15-209-2011.

[24] Abdo KS, Fiseha BM, Rientjes THM, Gieske ASM, Haile AT (2009) Assessment of climate change impacts on the hydrology of Gilgel Abay catchement in Lake Tana basin, Ethiopia. Hydrol Process 23: 3661–3669.

[25] George HL (1994) Modeling the effects of climate change on water resources - a review. Clim Chang 28: 159–177. doi:10.1007/BF01094105.

[26] MoWR (1998). bay River Basin Integrated Dev Master Plan Proj: 140.

[27] Mohamed Y, Hurk B Van Den, Savenije H, Bastiaanssen W (2005) Hydroclimatology of the Nile: results from a regional climate model. Hydrol Earth Syst Sci 9: 263–278. doi:10.5194/hess-9-263-2005.

[28] Setegn SG, Srinivasan R, Dargahi B, Melesse AM (2009) Spatial delination of soil erosion vulnerability in the Lake Tana Basin, Ethiopia. Hydrol Process 23: 3738–3750.

[29] Carter T, Hulme M, Lal M (1999) Guidelines on the Use of Scenario Data for Climate Impact and Adaptation Assessment. version 1.

[30] Mearns L, Giorgi F, Whetton P, Pabon D, Hulme M et al. (2003) Guidelines for use of climate scenarios developed from Regional Climate Model experiments.: 38.

[31] Wilby R, Hay L, Leavesley G (1999) A comparison of downscaled and raw GCM output: implications for climate change scenarios in the San Juan River basin, Colorado. J Hydrol 225: 67–91. doi:10.1016/S0022-1694(99)00136-5.

[32] Lines GS, Pancura M, Lander C (2006) Building climate change scenarios of temperature and precipitation in atlantic Canada using the Statistical Downscaling Model (SDSM).

[33] Wilby RL, Dawson CW (2007) Using SDSM version 4.1 SDSM 4.2; A decision support tool for the assessment of regional climate change impacts. User Manual. Leics.,: LE11 3TU, UK.

[34] Canada E (2009) HadCM3 Predictors: A2(a) and B2(a) Experiments. Available: http://www.cccsn.ec.gc.ca/?page=pred-hadcm3. Accessed 12 March 2009.

[35] Kim U, Kaluarachchi JJ (2009) Climate Change Impacts on Water Resources in the Upper Blue Nile River Basin, Ethiopia. JAWRA. J Am Water Resour Assoc 45: 1361–1378. doi:10.1111/j.1752-1688.2009.00369.x.

[36] Wilby R, Harris I (2006) A framework for assessing uncertainties in climate change impacts: Low-flow scenarios for the River Thames, UK. Water Resour Res 42: 1–10.

[37] Lieshout van M, Kovats R, Livermore M, Martens P (2004) Climate change and malaria: analysis of the SRES climate and socio-economic scenarios. Glob Environ Chang 14: 87–99. doi:10.1016/j.gloenvcha.2003.10.009.

[38] NMSA (2009) Metreological data. Agency: The Ethiopian National Metreological Services.

[39] Neitsch S, Arnold J, Kiniry J, Williams J (2005) Soil and Water Assessment Tool (SWAT) Theoretical Documentation, version 2005,. Temple, TX: Grassland Soil and Water Research Laboratory, Agricultural Research Service, Blackland Research Center, Texas Agricultural Experiment Station.

[40] Setegn SG, Srinivasan R, Melesse AM, Dargahi B (2010) SWAT model application and prediction uncertainity analysis in the Lake Tana Basin, Ethiopia. Hydrol Process 24: 357–367.

[41] Easton ZM, Fuka D, White E, Collick AS, Ashagre BB et al. (2010) A multi basin SWAT model analysis of runoff and sedimentation in the Blue Nile, Ethiopia. Hydrol Earth Syst Sci 14: 1827–1841. doi:10.5194/hess-14-1827-2010.

[42] Betrie G, Mohamed Y, Van AG, Srinivasan R (2011) Sediment management modelling in the Blue Nile Basin using SWAT. Hydrol Earth Syst Sci 15: 807–818. doi:10.5194/hess-15-807-2011.

[43] NRCS (2004) Estimation of Direct Runoff from Storm Rainfall chapter 10. Part 630 Hydrology: National Engineering Handbook. The U.S. Department of Agriculture (USDA). p. 79.

[44] Winchell M, Srinivasan R, Di Luzio MJ (2009) ArcSWAT 2.3.4. Temple, TX: Interface for SWAT2005.

[45] SRTM (2009) SRTM Data Selection Options. Available: http://srtm.csi.cgiar.org/SELECTION/inputCoord.asp. Accessed 10 February 2009.

[46] MoWR (2009) Spatial and hydrological data, The Federal Democratic Republic of Ethiopia, Minstry of Water. Resources.

[47] FAO (1995) World Soil Resources: An explanatory note on the FAO World Soil Resources Map at 1:25 000 00 scale. Rome.

[48] Refsgaard JC (1997) Parameterisation, calibration and validation of distributed hydrological models. J Hydrol 198: 69–97. doi:10.1016/S0022-1694(96)03329-X.

[49] Van Griensven A, Meixner T, Grundwald S, Bishop T, Diluzio M et al. (2006) A global sensitivity analysis tool for the parameters of multi-variable catchment models. J Hydrol 324: 10–23. doi:10.1016/j.jhydrol.2005.09.008.

[50] Nash JE, Sutcliffe JV (1970) River flow forecasting through conceptualmodels: Part 1. - A discussion of principles. J Hydrol 10: 282–290. doi:10.1016/0022-1694(70)90255-6.

[51] Bader D, Covey C, Gutowski W, Held I, Kunkel K et al. (2008) Climate Models: An Assessment of Strengths and Limitations.

[52] Santhi S, Arnold J, Dugas W, Srinivasan R, Hauck L (2001) Validation of SWAT model on a large river basin with point and nonpoint sources. J Am Water Resour Assoc 37: 1169–1188. doi:10.1111/j.1752-1688.2001.tb03630.x.

[53] IPCC (2007) Climate change 2007: Impacts adaptation and vulnerability - summary for policy makers Working Group II contribution to the Fourth Assessment Report of The Intergovernmental Panel on Climate Change.

[54] Setegne SG, Rayner D, Melesse AM, Dargahi B, Srinivasan R (2011) Impact of climate change on the hydroclimatology of Lake Tana Basin, Ethiopia. Water Resour Res 47: 13.

[55] IPCC (2007) Climate Change: The physical Science Basis. Contribution of Working Group I to the Fourth Assessment Report of the Intergovernmental Panel on Climate Change. Solomon SD UK, New York, NY, USA: Cambridge University Press.

[56] Maurer EP (2007) Uncertainty in hydrologic impacts of climate change in the Sierra Nevada, California, under two emissions scenarios. Clim Change 82: 309–325. doi:10.1007/s10584-006-9180-9.

[57] Fowler H, Blenkinsop S, Tebaldi C (2007) Linking climate change modelling to impacts studies: recent advances in downscaling techniques for hydrological. Int J Climatol 27: 1547–1578. doi:10.1002/joc.1556.

[58] Hawkins E, Sutton R (2010) The potential to narrow uncertainty in projections of regional precipitation change. Clim Dyn 37: 407–418.

[59] Giannini A, Biasutti M, Held IM, Sobel AH (2008) A global perspective on African climate. Clim Change 90: 359–383. doi:10.1007/s10584-008-9396-y.

[60] Hulme M, Doherty R, Ngara T, New M, Lister D (2001) African climate change : 1900 - 2100. Clim Res 17: 145–168. doi:10.3354/cr017145.

[61] Cook KH, Edward KV (2006) Coupled Model Simulations of the West African Monsoon System: Twentieth- and Twenty-First-Century Simulations. J Climatol 10: 3681–3703.

[62] Held IM, Delworth TL, Lu J, Findell KL, Knutson TR (2006) Simulation of Sahel drought in the 20th and 21st centuries. Proc Natl Acad Sci USA 103: 17891–6. PubMed: 16322101. 16322101 10.1073/pnas.0509057102 PMC1312412

[63] Hoerling M, Hurrell J, Eischeid J, Phillips A (2006) Detection and Attribution of Twentieth-Century Northern and Southern African Rainfall Change. J Climatol 19: 3989–4008. doi:10.1175/JCLI3842.1.

[64] Maurer EP, Duffy PB (2005) Uncertainty in projections of streamflow changes due to climate change in California. Geophys Res Lett 32: L03704. doi:10.1029/2004GL021462.

